# Construction of a sensitive and specific lead biosensor using a genetically engineered bacterial system with a luciferase gene reporter controlled by *pbr* and *cadA* promoters

**DOI:** 10.1186/s12938-020-00816-w

**Published:** 2020-10-19

**Authors:** Esmail Nourmohammadi, Saman Hosseinkhani, Reza Nedaeinia, Hoda Khoshdel-Sarkarizi, Mozhdeh Nedaeinia, Maryam Ranjbar, Neshat Ebrahimi, Zahra Farjami, Mohammad Nourmohammadi, Ali Mahmoudi, Mohammad Goli, Gordon A. Ferns, Majid Sadeghizadeh

**Affiliations:** 1 Department of Medical Biotechnology, Faculty of Medicine, Mashhad University of Medical Sciences, Mashhad, Iran; 2 Research Center of Advanced Technologies in Medicine, Torbat Heydariyeh University of Medical Sciences, Torbat Heydariyeh, Iran; 3 Department of Biochemistry, Faculty of Biological Sciences, Tarbiat Modares University, Tehran, Iran; 4 Pediatric Inherited Diseases Research Center, Research Institute for Primordial Prevention of Non-Communicable Disease, Isfahan University of Medical Sciences, Isfahan, Iran; 5 Applied Physiology Research Center, Isfahan University of Medical Sciences, Isfahan, Iran; 6 Advanced Materials Research Center, Department of Materials Engineering, Najafabad Branch, Islamic Azad University, Najafabad, Iran; 7 Laboratory of Cedars-Sinai Medical Center, Los Angeles, CA USA; 8 Department of Health Research Center, Mashhad University of Medical Sciences, Mashhad, Iran; 9 Department of Food Science and Technology, Isfahan (Khorasgan) Branch, Islamic Azad University, Isfahan, Iran; 10 Brighton and Sussex Medical School, Division of Medical Education, Falmer, Brighton, BN1 9PH Sussex UK; 11 Department of Molecular Genetics, Faculty of Biological Sciences, Tarbiat Modares University, Tehran, Iran

**Keywords:** Lead, Bacterial biosensor, *Pbr* promoter, Luciferase, *cadA* promoter

## Abstract

**Background:**

A bacterial biosensor refers to genetically engineered bacteria that produce an assessable signal in the presence of a physical or chemical agent in the environment.

**Methods:**

We have designed and evaluated a bacterial biosensor expressing a luciferase reporter gene controlled by *pbr* and *cadA* promoters in *Cupriavidus metallidurans* (previously termed *Ralstonia metallidurans*) containing the *CH34* and *pI258* plasmids of *Staphylococcus aureus*, respectively, and that can be used for the detection of heavy metals. In the present study, we have produced and evaluated biosensor plasmids designated *pGL3-luc/pbr* biosensor and *pGL3-luc/cad* biosensor, that were based on the expression of luc+ and under the control of the *cad* promoter and the *cadC* gene of *S. aureus* plasmid *pI258* and *pbr* promoter and *pbrR* gene from plasmid *pMOL30* of *Cupriavidus metallidurans*.

**Results:**

We found that the *pGL3-luc/pbr* biosensor may be used to measure lead concentrations between 1–100 μM in the presence of other metals, including zinc, cadmium, tin and nickel. The latter metals did not result in any significant signal. The *pGL3-luc/cad* biosensor could detect lead concentrations between 10 nM to 10 μM.

**Conclusions:**

This biosensor was found to be specific for measuring lead ions in both environmental and biological samples.

## Background

Ecological heavy metal pollution is a common problem that can lead to damage to human health [1]. These heavy metal pollutants may lead to environment damage and harmful ecological outcomes [2], and hence the development of sensitive, efficient, rapid and cost-effective methods is necessary to screen for the presence of these harmful metals in the environment. Lead (Pb) is a toxic heavy metal that is extensively utilized around the world [3, 4]. It has been estimated that the world production of lead is more than 3 million tons per year. It causes widespread environmental contamination in the air, water, soil, and food [5]. This element can enter human bodies as well as animals, affecting the integrity of the food chain; in fish it can accumulate in the bone, liver, gills, kidney, ovary, and muscle [6].

Environmental lead may result in high blood concentrations and an increase in vascular endothelial growth factor (VEGF) [7, 8], and can lead to neurological and cardiovascular complications [9]. The reproductive system may also lead to developmental disorders in children [10–13]. Lead can cross the placenta and cause damage to the developing fetal nervous system [14].

The assessment and monitoring of environment heavy metal contamination is therefore very important to prevent harm to human health. Currently, classical analytical methods, such as spectrometry, FIAAS (Flow injection atomic absorption spectrometry), ion chromatography, and electrochemical techniques, are the main methods used for measuring environmental heavy metals pollution. The main disadvantage of these methods is the necessity for sample digestion under high temperature and pressure, or acidic conditions in which metal ions in solution are released [15]. In any case, the specified apparatus is exceptionally expensive, requires appropriately trained analysts, and it may take days or weeks to get results from a specialist laboratory. Therefore, simpler methods for evaluating heavy metals are required. More importantly, heavy metals are found to be present in the biological systems either in bioavailable/toxic or non-available/non-toxic forms, and current measuring methods are unable to distinguish between toxic and non-toxic fractions of these elements [16], and these methods are both time-consuming and costly [17]. Biosensors have been developed that are an effective alternative to conventional detecting systems. These may be highly sensitive and simple to use [18]. Cell-based biosensors are biological sensors that contain a reporter gene under the control of a promoter that is sensitive to the presence of an agent, such as environmental contaminants that include heavy metals. Biosensors are used in various designs with different reporters and promoters. At low concentration of heavy bioavailable metals, bioluminescence signals are likely to be suitable [19, 20]. Hence while classical analytical techniques can detect metal ion contaminants in environmental samples with excellent precision, they are complex and costly and do not differentiate between the unavailable and bioavailable fractions. An approximate of the bioavailable fraction is significant in bioremediation, waste dumping, waste-treatment optimization and the evaluation of environmental impact [21–27]. Cell-based biosensors can also be applied to monitoring bioavailable concentrations of heavy metals and piezoelectric biosensors as enzyme-based electrochemical biosensors [28–30]. One of the most obvious advantages of this method is the ability to measure the bioavailable heavy metal at very low concentrations. It is also a cost-effective and time saving method [18]. In these biosensors, the expression of a reporter gene is controlled by a promoter, such as the *pbrR* promoter in the *pMOL30* plasmid of *Cupriavidus metallidurans* *CH34* and *cadC* promoter in *pI258* plasmid of *Staphylococcus aureus* (*S. aureus*) that is sensitive to heavy metals. Most of these promoters originate from bacteria that have resistance systems against heavy metals [31, 32]. In this study, we have designed and evaluated luciferase reporter gene expression of bacterial biosensor under the control of *pbr* and *cadC* promoters in *Cupriavidus metallidurans CH34* and *pI258* plasmids of *Staphylococcus aureus*, respectively, for the measurement of lead.

## Results

### Sequencing

**Fig. 1:**
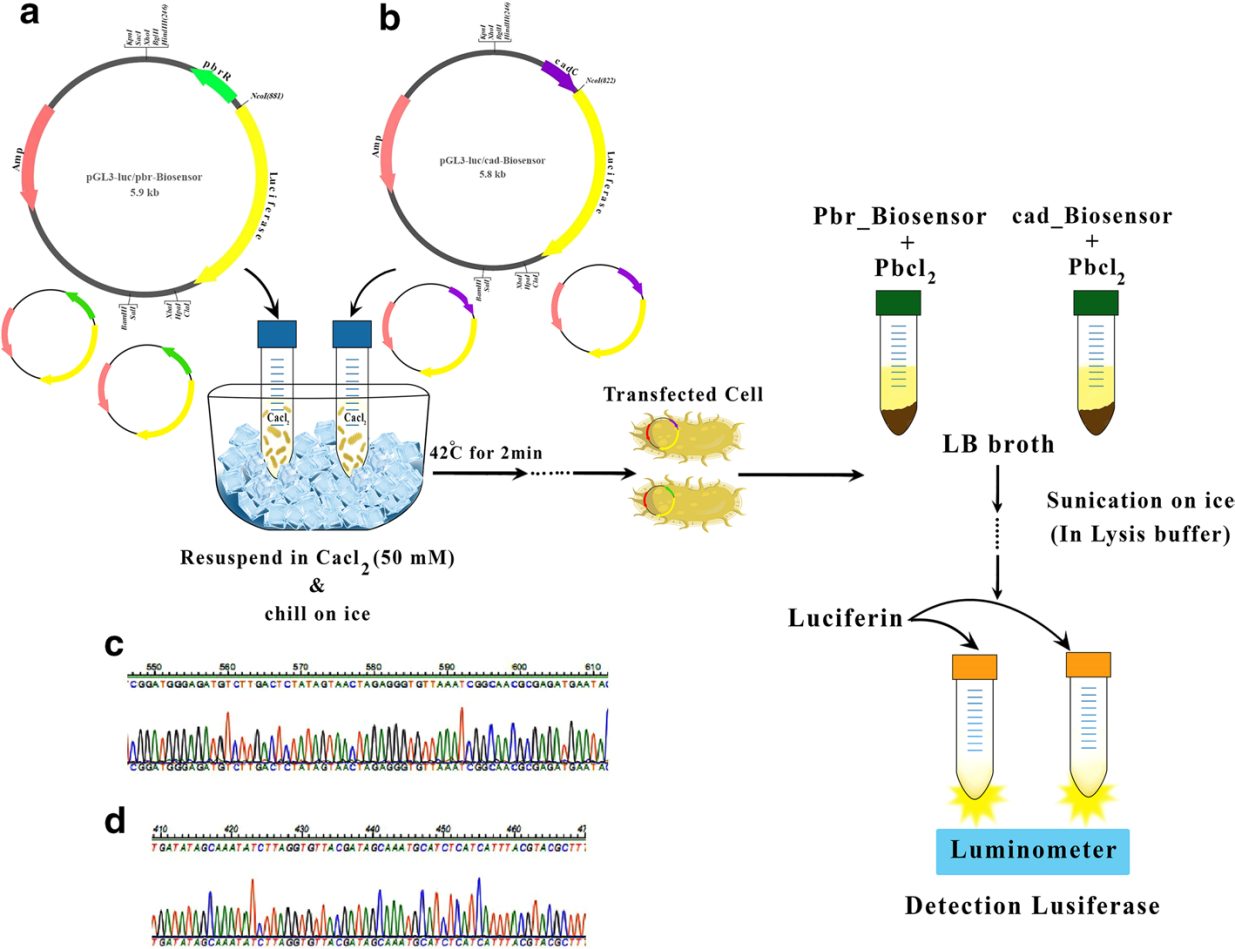
Simplified schematic representation of the *E. coli* strain DH5α transfection. **a** Recombinant plasmid (*pGL3-luc/pbr* biosensor)*.* **b** Recombinant plasmid (*pGL3-luc/cad* biosensor). *pGL3-luc/pbr* biosensor and *pGL3-luc/Cad* biosensor were transferred to the *E. coli* strain *DH5α* using the chemical method of CaCl_2_ and then were screened using selective plates containing antibiotic ampicillin. **c** Sequencing and integrity of synthesis sequence. **d** *PGL3-luc/pbr* biosensor *pGL3*-luc/cad biosensor. The promoter region was sequenced in the received plasmid

**Fig. 2:**
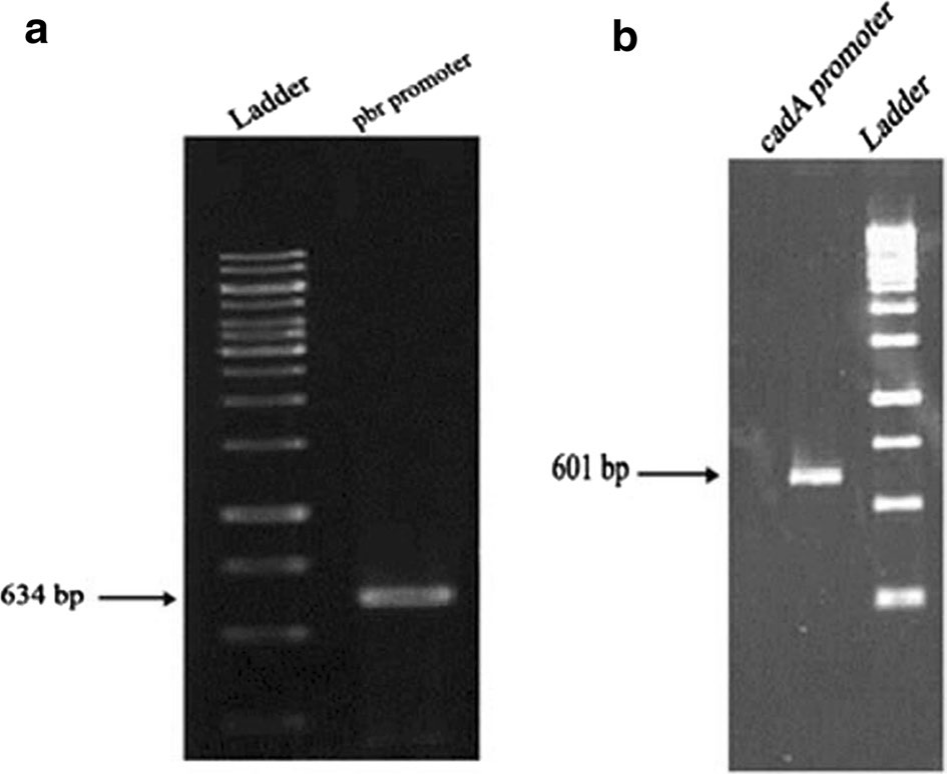
**a** The proliferation region of the *pbr* promoter with 634 bp. **b** *cadA* promoter with 601 bp. The promoter sequence and regulatory gene were amplified with 634 bp for *pbr* and 601 bp for c*adA*. 1 kb DNA Ladder (Containing 14 linear double-stranded DNA fragments)

In order to ensure the integrity of the sequencing, the promoter region was sequenced in the modified plasmid (Fig. 1c, d). PCR was performed using primers designed for the *pbr* and *cadA* promoters, and the promoter sequence and regulatory gene were amplified with 634 bp for *pbr* and 601 bp for *cadA* (Fig. 2).

### Biosensor activity of *pGL3-luc*/*pbr*

The expression of the luciferase gene, in the presence of different concentrations of lead, showed that 1 μM of lead was the lowest concentration that could stimulate the promoter and could be distinguished from the basal expression of luciferase, and the highest measureable expression was seen at 100 μmol/L. A good biosensor should have two characteristics: specificity and sensitivity. According to the data obtained from our experiments, this biosensor had a high specificity, and luciferase gene was only expressed in the presence of lead.

### Biosensor specificity for lead in the presence of different concentrations of zinc (ZnCl_2_), tin (SnCl_2_) and cadmium (CdCl_2_)

**Fig. 3:**
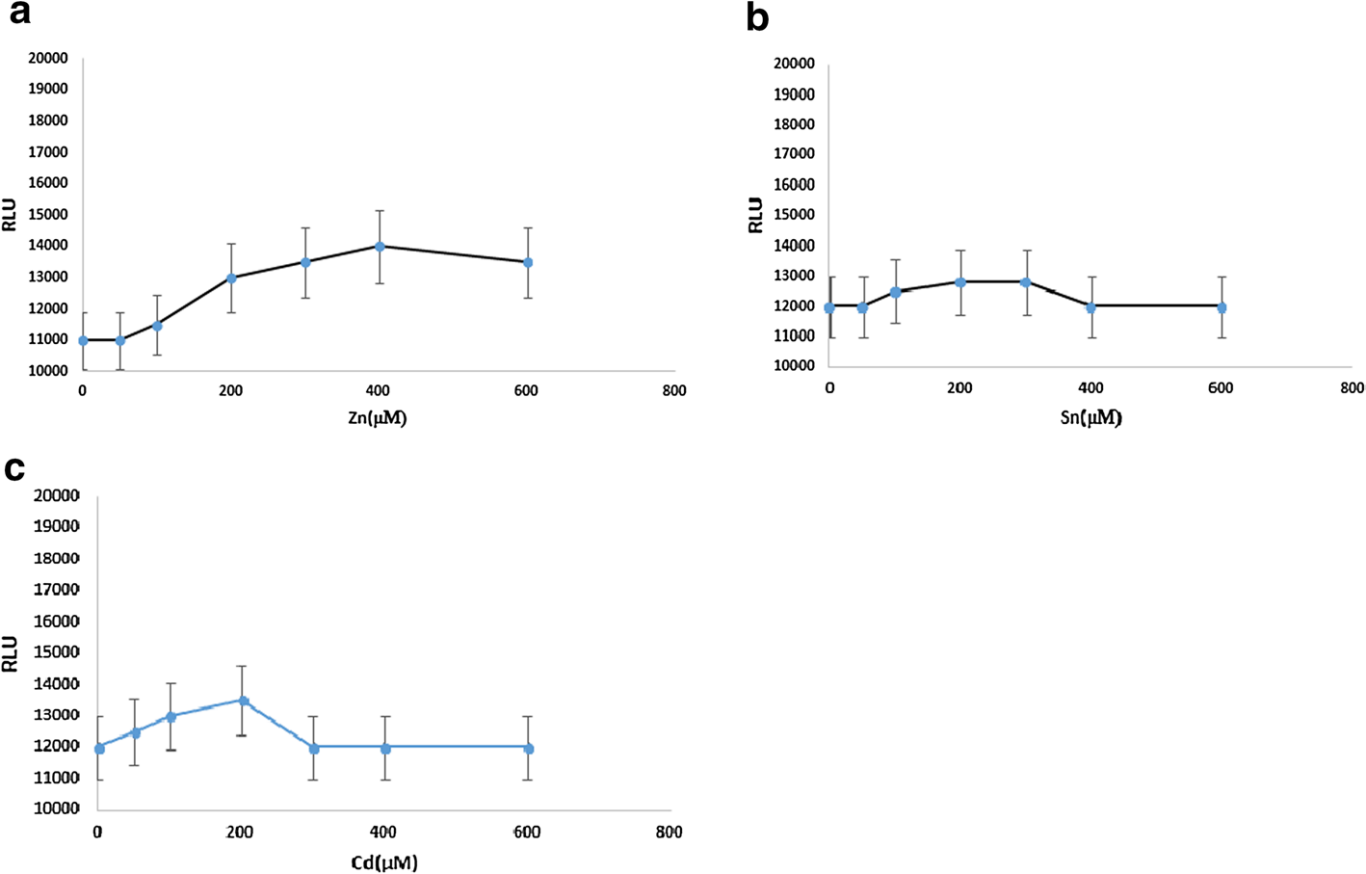
Expression of luciferase gene in different concentrations of zinc, tin and cadmium**.** Heavy metal had no effect on the stimulation of the *pbr* promoter. The *pbr* promoter is specific to lead, and other heavy metals such as **a** zinc (Zn), **b** tin (Sn) **a** and **c** cadmium (Cd), do not activate the promoter and significant expression of a reporter gene

The biosensor was cultured in the presence of different concentrations of zinc, tin and cadmium, and did not stimulate the *pbr *promoter and expression of the reporter gene (Fig. 3). In Fig. 3, we aimed to show that the pbr promoter is specific to lead, and other heavy metals such as zinc (Zn) (Fig. 3a), tin (Sn) (Fig. 3b) and cadmium (Cd) (Fig. 3c) do not activate the promoter and significant expression of a reporter gene. Data obtained from the expression of the luciferase gene in the presence of various concentrations of tin, zinc and cadmium, indicated that these heavy metals did not stimulate the *pbr* promoter.

### Biosensor activity in the presence of different concentrations of lead (PbCl_3_)

**Fig. 4:**
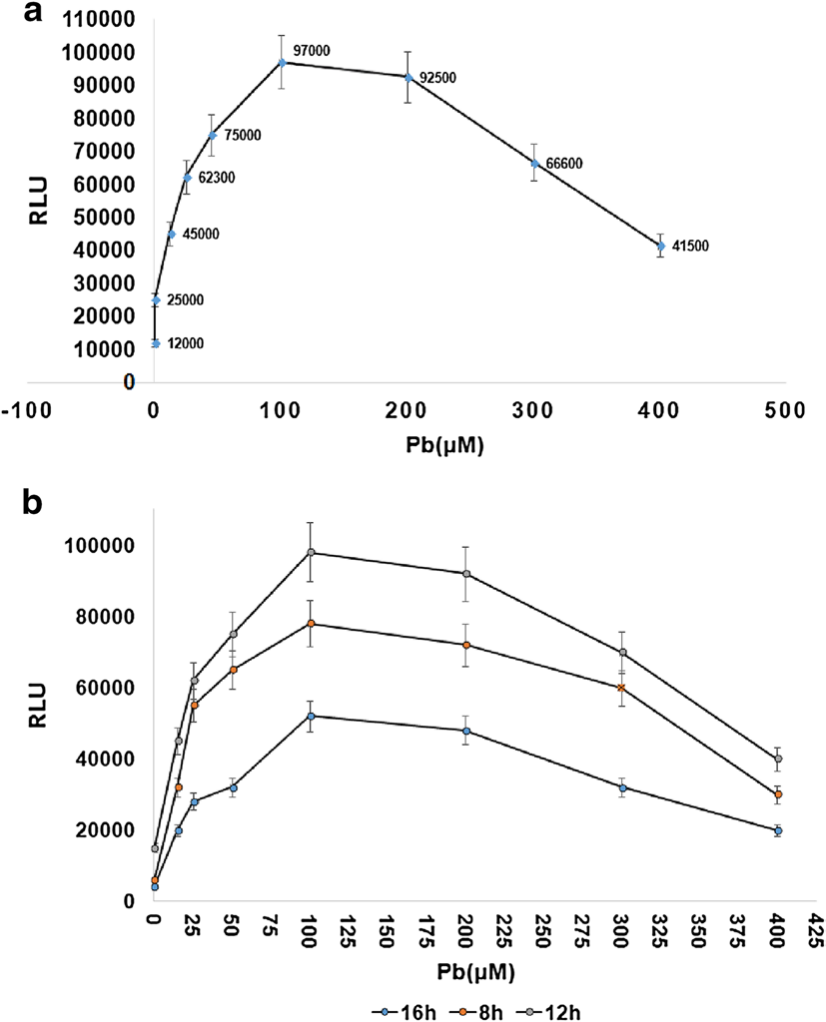
**a** Luciferase expression in different concentrations of lead. The expression of luciferase was decreased with a slight gradient from 100 to 200 micro molar. Luciferase gene expression was shown to be present in different concentrations of lead. Luciferase expression is increased by increasing lead, but from a concentration level of 100 μM onwards, the expression rate decreases due to the toxic effects of lead, and this decrease intensifies from a concentration level of 200 μM onwards. Relative luminescence units (*RLU*). **b** The expression of *pGL3-luc/pbr* biosensor reporter gene at different times. The biosensor was treated in the presence of lead in three time periods of 8, 12, and 16 h, and the expression gene of the reporting gene was evaluated to obtain the appropriate time for the treatment of the biosensor with lead. The expression time is low at 8 h, and the expression time is reduced at 16 h due to the overload of the biosensor bacteria and the toxic effects of lead metal

Lead was the only metal that stimulated the *pbr* promoter. In the absence of lead, the regulator gene prevents the promoter from activation. Lead ions bind to the regulator gene and inhibits its binding to the operator. As a result, the promoter is activated and the luciferase is expressed. The minimum detectable concentration of this biological sensor was approximately 1 µM and a maximum is 100 μmol/L. The expression of luciferase was no longer linear for value of lead from 100 to 200 μmol/L (Fig. 4a).

### The expression of *pGL3-luc*/ *pbr* biosensor reporter gene at different times

**Fig. 5:**
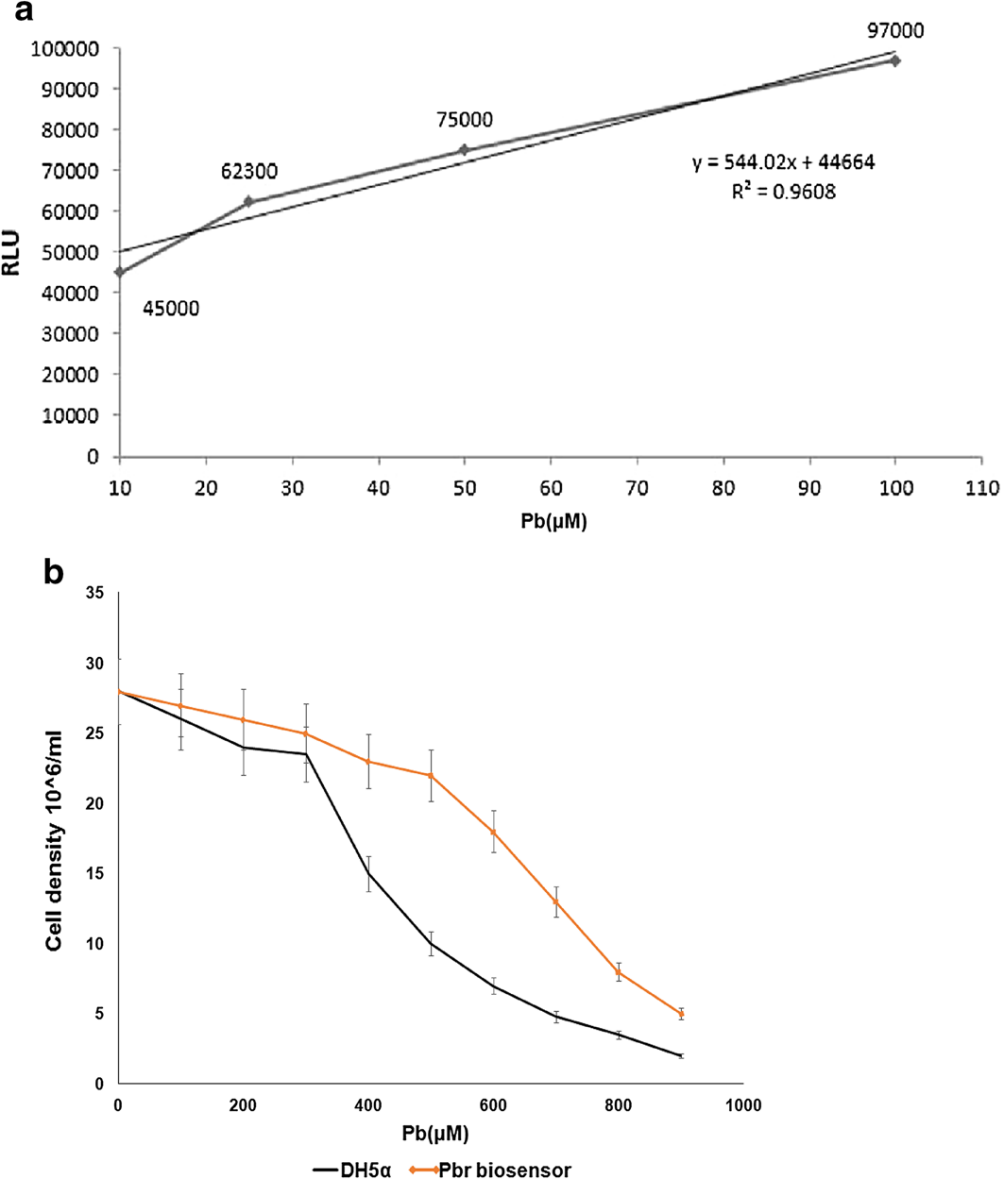
**a** Linear expression ranges of luciferase in the presence of lead with regression coefficient *R*^2^ = 0.960. The maximum expression of the luciferase gene was 12 h. Luciferase expression (in the range of 10–100) is linear with high regression, and the sensor in this range can detect the presence of lead with a lower error coefficient. **b** Difference in the growth rate of *pGL3-luc/pbr* biosensor compared to *E. coli* strain *DH5α*. Resistance may be related to the* pbrR *regulatory gene. The presence of *pbrR* as regulator gene, in the positions of the binding of the lead ion, makes the promoter somewhat resistant to lead toxicity and more resistant to plasmid-free bacteria

In order to identify the appropriate time for biosensor growth, a biosensor was cultured at different concentrations of lead for different durations (Fig. 4b). The maximum expression of the luciferase gene was at 12 h (Fig. 5a).

### The difference in the growth rate of *pGL3-luc*/ *pbr* biosensor compared to *E. coli* strain DH5α

The sensor bacteria had a recombinant plasmid containing the *pbr* promoter region and the *pbrR* regulatory gene. These bacteria have a greater resistance to lead than *E. coli DH5α* without plasmid. This resistance may be related to the *pbrR* regulatory gene (Fig. 5b). The resistance genes to heavy metals have heavy metal-binding motifs, they can limit the toxicity of these metals inside the cell, because of these proteins, the relative resistance of the cell to heavy metals.

### The activity of *pGL3-luc/cad* biosensor at different concentrations of lead

**Fig. 6:**
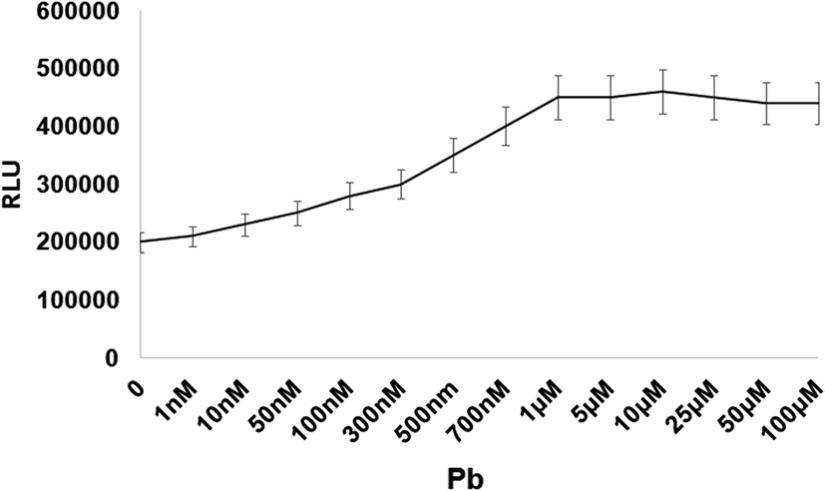
Expression of luciferase gene in different concentrations of lead. Luciferase gene expression controlled by *cad *promoter and *cadR* regulatory gene in the presence of different lead concentrations. Low levels of lead concentrations in about 10 nM cause significant expression of the reporter gene

**Fig. 7:**
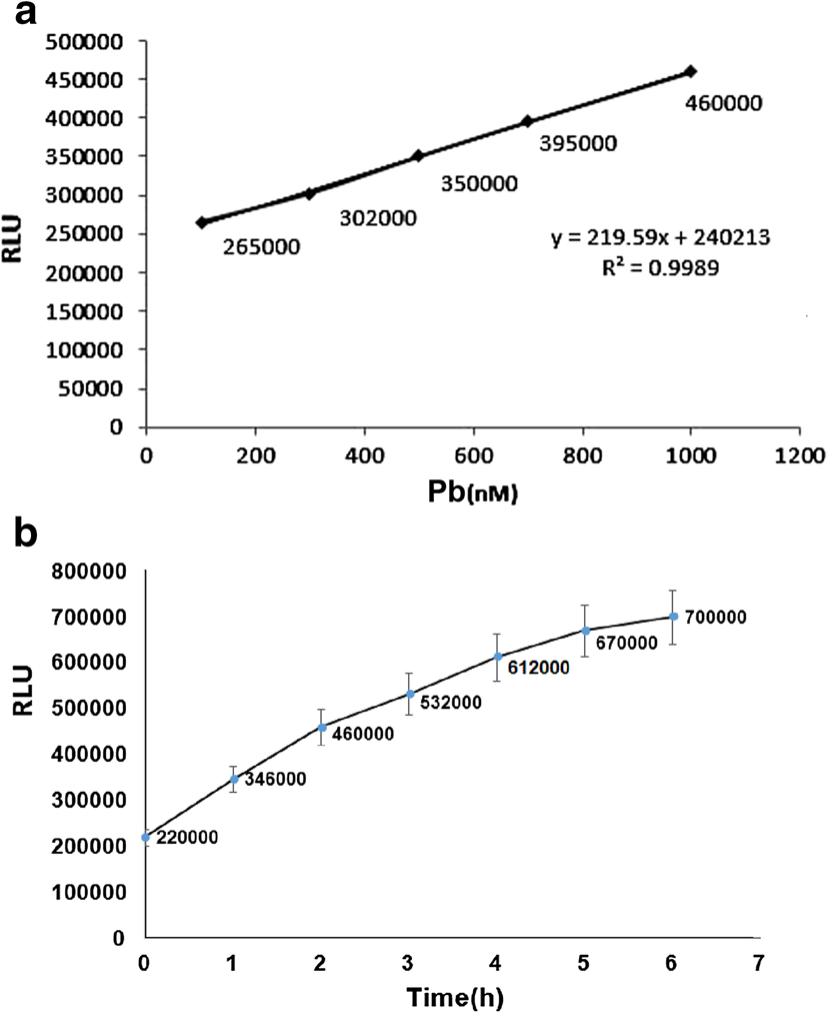
**a** Linear expression ranges of luciferase expression between 100 and 1000 nM concentrations of lead. **b** The expression of luciferase at different times at 1 μM Pb concentration. During 2 h, the amount of expression is high enough to measure luciferase, in biological sensors; the pollution is measured at low rates

The lowest and highest concentrations of lead that could stimulate the expression of the reporter gene were 10 nmol/L and 10 μmol/L, respectively (Figs. 6 and 7a).

### Expression of the luciferase gene in the presence of 1 μmol concentration of lead at different times

The sensor bacteria were incubated at 0.2 OD (1 μmol/L concentration) for different times in the incubator. The expression of luciferase was measured at different times (Fig. 7b). As shown in Fig. 7b, the concentration of 1 μM lead can induce luciferase expression. The degree of expression increased with time, with measureable change in luciferase levels by 2 h measure, and in biological sensors pollution is usually measured at low rates, we chose 2 h for culture of the *pGL3-luc/cad *biosensor.

## Discussion

Because of global industrialization and various geochemical processes, heavy metals and metalloids are the natural parts of an ecosystem which approach the food chain. Only a small rise in these non degradable pollutants' concentration creates a serious danger to organisms [33]. Heavy metals, such as organic pollutants, are not degradable but can be transformed to exist in less toxic form. Microbes are the cheap weapon since they change quickly to overcome heavy metal pressure by creating appropriate survival techniques, like sequestration or active metal transport [34]. The key sources of pollutants in water quality are heavy metal ions like Pb^2+^ and Cd^2+^. Recently, full-cell detection has been extensively investigated to use genetically modified bacteria to detect the existence of heavy metal ions in water or soil. Whole-cell sensors require simple sample preparation and can continually sense metal contaminants in the cell culture environment in comparison with main cell-free techniques like immunosensor and electrochemical sensor [35]. It was indicated that strain *C. metallidurans CH34* is facultative chemolitho-autotrophic β-proteobacterium in the *Burkholderiaceae/order Burkholderiales* family. It was shown to be heavily resistant to Zn^2+^, Cd^2+^, Ni^2+^, AsO_43−_CrO_42−_, Hg^2+^, Ag^+^, Cu^1+^/^2+^, Pb^2+^, and Co^2+^ [36]. The whole-cell biosensors have been successfully produced using fluorescent and enzymatic reporters as elements of signal-output based on the natural pbr operon [37]. Biosensor is an analytical tool used to detect the targeted compounds easily and quickly. Furthermore, by *cadC* gene expression and promoter cad of *S. aureus* plasmid pI258 with GFP gene in *E. coli DH5α*, a whole-cell biosensor was developed for detecting toxic cadmium metal ions. The response time was 15 min, with a 10 μg/L detection limit. Luciferase reporter gene has also been expressed based on similar promoter and resistance determinant in *S. aureu*s *RN 4220* and *Bacillus subtilis* *BR151*. Cadmium, lead and zinc were detected by the resultant luminescent sensor [38].

There are several advantages to using bacterial biosensors, including speed, simplicity and cost. Biological sensors containing *cadA* and *pbr* promoter regions have been designed by other researchers, the optimization of this cell biological sensor with ability to measure lead comparing the *cadA* and *pbr* promoters in a bioassay system was evaluated in this study. The use of biosensors or biological cell sensors containing a reporter gene controlled by promoters susceptible to the heavy metal ions can provide an efficient method to trace particular pollutants in the environment and in a biological solution [39]. The present study assessed a biosensor system for detecting lead ions through construction of a luminescent bacterial sensor containing the *luc*^+^ regulated by the cad promoter and *ca*d*C* gene in plasmid *pI258* of *S. aureus* and the *pbr* promoter and *pbrR* gene in *pMOL30* plasmid of *Cupriavidus metallidurans*. Pb-specific bacterial biosensors were formerly defined using reporter genes including *lacZ*, *lux, *and *luc* in the transcription fusion constructs [40–42]. In our study, the luciferase reporter gene was used. Luciferases, as a set of heterogeneous enzymes, are able to produce light as a byproduct of catalyzing reactions. They are reporter genes extensively used by prokaryotic and eukaryotic organisms due to their high sensitivity and ease of detection.The quantification of the emitted light, i.e., bioluminescence,is of great importance; it can also be measured using a liquid scintillation counter, a luminometer, or even a X-ray film [41]. It was concluded that a *pGL3-luc*/*pbr* biosensor can detect Pb^2+^ in the range of 1–100 μM using the expression of firefly luciferase as a detector system, and is highly specific, with no expression of reporter in the presence of other metals such as Sn^2+^, Ni^2+^, Cd^2+^ are present. Moreover, this biosensor was 50 times more sensitive when compared with the previous biosensors reported by Chakraborty et al. [32]. The *R. metallidurans* *CH34* strain has several resistance systems that can reduce the concentration of toxic substances to their non-toxic levels. A highly specific system for resistance to lead is known in plasmid *pMOL30 *[43]. It effectively reduced the concentration of lead ions and is equipped with specific mechanisms for the transfer and separation of lead. The *pbr* operon includes *pbrA*, *pbrB*, *pbrC* and *pbrD* genes in which *pbrD* has a role as a chaperone to accumulate lead in the cell and *pbrA* eliminates lead ions [43]. Our results show that the *pGL3-luc*/*pbr* biosensor is not expressed in the presence of cadmium, zinc, or tin, indicating high sensitivity and specificity of the designed system for lead detection. One of the most important heavy metal transfer systems in *S. aureus* is located in the plasmid *pI258*. The plasmid has an operon *cadA* that encodes an ATPas of type P, which causes resistance to metals such as cadmium, lead, zinc, copper, and tin. The expression of the *cadA* operon is controlled by the *cadC* homodimeric protein. This protein is able, in a binary manner, to bind to the promoter and metal ions, such as cadmium, lead, zinc, and tin. The *cad* belongs to *ArsR/SmtB*, a regulating protein family [44]. In our study, the luciferase gene was used as a reporter and *E. coli* strain of *DH5α* as a host. Our results showed that the *pGL3-luc*/*cad* biosensor can detect at least 10 nM of lead and the lead toxicity was not observed until a concentration of 300 μM. However, the maximal expression of the reporter gene was performed at 10 μM. Our results are supported by the report of Liao et al. that showed the regulating role of cad promoter and the *cadC* gene in plasmid *pI258 *of *S. aureus*, the fluorescence emission was intensified with increasing Cd(II), Pb(II), and Sb(III) ions concentrations [45]. For Pb (II), just like our result in *pGL3-luc/cad* biosensor, to induce GFP expression significantly, 10 nM was the low, and 10 μM was the maximum concentration of lead that induced significantly GFP expression [45]. The metallo-regulatory α_3_N thiolate-rich site in *cadC* displays a practical selectivity for larger, softer heavy metal like Pb(II), Cd(II), although smaller boundary metal ions such as Zn(II) accommodated [46]. One of the limitations of this method is that bacterial biosensors require the necessary conditions for bacterial growth to operate, and the graphs are based on solving different concentrations of heavy metals in a bacterial culture medium. Therefore, to measure the amount of heavy metals in an unknown environment, it is necessary to optimize the biosensor in the new environment, which would itself require evaluation.

## Conclusion

Our results show that the maximum expression of reporter gene was found in the presence of 100 μM of Lead in *pGL3-luc*/*pbr *biosensor and 1 μM of lead in pGL3-*luc/cad* biosensor. In this study, the specificity and sensitivity of the two heavy metal susceptive probes, *pbr* and *cadA,* were investigated. Sensors containing these two promoter regions were able to detect the concentration of lead between 1–100 μM and 10 nM to 10 μM of lead, respectively. For other heavy metals such as mercury, copper, nickel, manganese, zinc and cadmium, different biological sensors can be made and their presence in the environment can be measured with very high accuracy. To determine the accuracy of biosensors, a standard curve of luciferase gene expression was plotted at different lead concentrations. The standard curve was constructed from triplicates values, we evaluated the accuracy of the biosensor with the specific concentrations that we had obtained from lead metals. By developing these sensors, the time required to identify environmental pollution can be minimized.

## Methods

### Chemicals

Analytical reagents, media and buffer solutions like TBE–EDTA buffer (Tris–borate–ethylenediaminetetraacetic acid), NaOH (Sodium hydroxide), CaCl_2_ (Calcium chloride), boric acid, Tris base, and agarose were all purchased from Merck (Germany). Fermentas (Lithuania) supplied the restriction endonucleases *Nco1* and *Hind3*, *T4* DNA ligase, and molecular ladder 10,000-300 bp. We also supplied the DNA polymerase (TaKaRa LA Taq® DNA Polymerase), dNTP and MgCl_2_ from Takara (Beijing, China). In addition, the plasmid extraction kit and primers were brought from Bioneer (Seoul, South Korea).

### Construction of biosensor plasmid

*pMOL30* (X71400 AJ278984) and *PI258* (GQ900378.1) containing the *pbrR* gene (634 bp) and *CadC* gene (601 bp) (Accession number: *pbrR*: WP_003103716.1and *CadC*: WP_000726009, respectively, were synthesized and supplied by Millegen company. To ensure the accuracy of synthesized plasmid, the promoter region was sequenced. *PGl3*-control as a vector containing the Luciferase gene and *E. coli* strain *DH5α* as the host were used in our study. To obtain a large amount of *pMA-T* plasmid (a synthetic plasmid) which contains p-promoter sequences and the regulatory gene was sent to MilliGen, after evaluation at the NCBI site, for the synthesis of sequences. Synthesized sequences consisted of both *pbr*_*pMA-T* plasmids containing the promoter sequence of the *pRR* operon and the *pbrR* regulator gene including; *cadA pMS-RQ-Bs* plasmid containing the promoter region of the *cadAp* and the *cadA* gene regulating gene), it was cloned to *E. coli* host. Afterwards, *pMA-T* was extracted using plasmid extraction kit, and its quantity and quality were both examined by spectrophotometry and agarose gel, respectively, before they got digested by* HindIII* and *NcoI*. The promoter regions with the regulator genes were also purified from the gel electrophoresis. The received sequence and *pGL3*-control vector were cut using the same restriction enzyme (*Nco1* and *Hind3*) and ligation reaction at 37 °C for 3–4 h with ligase enzyme. The firefly luciferase gene was placed under the control of the received promoter sequences and recombinant plasmids of *cad* and *pbr* promoters were named *pGL3-luc*/*pbr* biosensor and *pGL3-luc/Cad* biosensor, respectively. Recombinant plasmids *pGL3-luc*/*pbr* biosensor (Fig. 1a) and *pGL3-luc/Cad* biosensor (Fig. 1b) were transferred to the *DH5α* bacteria using the chemical method of CaCl2 and then were screened using selective plates containing antibiotic ampicillin. After plasmid extraction, PCR was performed to detect colonies containing the promoter region of *pbr* and *cadA* using primers designed for the cloned fragments. After these processes, recombinant plasmids were used to evaluating and measuring different concentrations of heavy metals.

### Culture of bacteria and measuring biosensor activity of luciferase enzyme

To study the efficiency of promoters the detection of heavy metals, a luciferase enzyme measurement performed in the presence of lead and other heavy metals such as tin, zinc and cadmium. In this process, *E. coli* stains carrying *pGL3-luc/Cad* biosensor and *pGL3-luc*/*pbr* biosensor were cultured in Luria–Bertani (LB) broth that contained 100 µg/mL ampicillin at 37˚C, overnight. Then 50 µl from overnight grown culture of *pGL3-luc*/*pbr* biosensor for 12 h and *pGL3-luc/Cad* biosensor with optical density (OD_600_) 0.8 for 2 h were cultured in the presence of heavy metals at different concentrations [47]. Next, the culture was centrifuged at 5000 RPM  for 10 min at 4 °C metals for bacterial sedimentation. Then the medium was removed, and lysis buffer was added to the plate and sonicated at low temperature. Then, the amount of luciferase expression was measured by a luminometer (Berthold Company).

### Statistical analysis

All the experiments were repeated at triplicate to minimize error. The Student’s *t*-test and one-way analysis of variance (ANOVA) were used to compare the statistical significance between the two groups and each group was compared with the baseline through the same method. Statistical significance was set at *P ≤ 0.05. Data are shown as mean values ± standard deviation (SD). Linear regression was used to model the standard curve. Analysis of data was performed using SPSS version 22 statistical software (IBM, Chicago, IL, USA).

## Data Availability

All data generated or analyzed during this study are included in this published article.

## Abbreviations

Pb: Lead
Zn: Zinc
Sn: Tin
Cd: Cadmium
VEGF: Vascular endothelial growth factor
FIAAS: Flow injection atomic absorption spectrometry
LB: Luria–Bertani
RLU: Relative luminescence units
SD: Standard deviation
